# 3D variable flip angle T1 mapping for differentiating benign and malignant liver lesions at 3T: comparison with diffusion weighted imaging

**DOI:** 10.1186/s12880-022-00873-8

**Published:** 2022-08-18

**Authors:** Fei Wang, Qing Yang, Yupei Zhang, Jun Liu, Mengxiao Liu, Juan Zhu

**Affiliations:** 1 Department of Medical Imaging, Anqing Hospital Affiliated to Anhui Medical University, 352 Renmin Road, Anqing, 246000 China; 2 Siemens Healthcare Ltd., Shanghai, 201318 China

**Keywords:** MRI, Focal liver lesions, T1 mapping, Diffusion weighted imaging

## Abstract

**Background:**

Different methods have been used to improve the imaging diagnosis of focal liver lesions (FLL). Among them, magnetic resonance imaging (MRI) has received more attention since it provides significant amount of information without radiation exposure. However, atypical imaging characteristics of FLL on MRI may complicate the differential diagnosis between benign and malignant FLL. This study aimed to compare the diagnostic value of T1 mapping and diffusion-weighted imaging (DWI) for differentiating of benign and malignant FLLs.

**Methods:**

This retrospective study enrolled 294 FLLs, including 150 benign and 144 malignant lesions. Whole liver T1 mapping sequences were obtained before and 2 min after the administration of Gd-DTPA to acquire native T1 and enhanced T1 and ΔT1%. Additionally, DWI sequence was conducted to generate apparent diffusion coefficient (ADC) maps. These quantitative parameters were compared using one-way analysis of variance, and the diagnostic accuracy of T1 mapping and ADC for FLLs was calculated by area under the curve (AUC).

**Results:**

Significant differences were observed regarding the native T1, enhanced T1, ΔT1%, and ADC between benign and malignant FLLs. Furthermore, the sensitivity and specificity of the parameters are as follows: native T1 0.797/0.702 (cut off value 1635.5 ms); enhanced T1, 0.911/0.976 (cutoff value 339.2 ms); ΔT1%, 0.901/0.905 (cutoff value 70.8%); and ADC, 0.975/0.952 (cutoff value 1.21 × 10^−3^ mm^2^/s). The ideal cutoff values for native T1 and ADC in identifying cyst and haemangioma were 2422.9 ms (AUC 0.990, *P* < 0.01) and 2.077 × 10^–3^ mm^2^/s (AUC 0.949, *P* < 0.01), respectively, with a sensitivity and specificity of 0.963/1 and 0.852/0.892, respectively. ADC was significantly positively correlated with T1 and ΔT1%, and significantly negatively correlated with enhanced T1.

**Conclusion:**

The 3D Variable flip angle T1 mapping technique with Gd-DTPA has a high clinical potential for identifying benign and malignant FLLs. The enhanced T1 and ΔT1% values have similar diagnostic accuracy compared with DWI in evaluating FLLs. Native T1 shows better performance than DWI in distinguishing benign liver lesions, specifically, cysts, and haemangioma.

## Introduction

The rapid development and widespread use of medical imaging technologies have led to a continuous increase in the clinical detection rate of focal liver lesions (FLLs), which, in turn, has led to the use of different methods to improve the diagnostic accuracy of FLLs, including ultrasonic enhancement [1], ultrasonic elastography [2], dual-energy computed tomography [3], and magnetic resonance imaging (MRI) [4–8]. Of these methods, MRI has received more attention because it can provide a wide range of multi-contrast structural and functional information without radiation exposure. Conventional MRI commonly uses T2- (T2WI)and contrast-enhanced T1-weighted images (T1WI) to differentiate between benign and malignant FLLs. As a functional technique, diffusion-weighted imaging (DWI) with apparent diffusion coefficient (ADC), its quantitative compartment, has similarly shown its effectiveness for identifying FLLs. However, this method has some disadvantages. First, respiratory motion causes difficulties in ensuring DWI quality of the upper abdominal area, even with various gating techniques. Second, cancerous cells surrounded by other solid liver lesions, such as focal nodular hyperplasia (FNH) and metastases (MET), are difficult to identify using ADC [9]. Furthermore, for patients with cysts and haemangiomas (HEM), ADC differential diagnosis also has limitations or controversies [10].

For dynamic contrast-enhanced MRI (DCE-MRI), the standard method used to diagnose FLLs is to focus on the signal intensity (SI) change pre- and post-contrast administration [11]. Therefore, subjectivity may be unavoidable during visual evaluations of liver lesions using contrast-enhanced MRI. T1 relaxation time is an inherent tissue characteristic and can be used for non-invasive visualisation of pathological changes. Native and enhanced T1 and extracellular volume can be acquired and calculated with a contrast agent, which reflects the absorption content of gadolinium in the lesion and the surrounding tissue. Variable flip angle (VFA) is currently a popular method for T1 mapping because of its significant coverage of the body, which allows acquisition of high-spatial resolution T1 values within a single breath-hold. In addition, the accuracy of VFA is improved by applying a simple B1 correction procedure, making it more feasible for clinical practice [12]. The administration of a liver-specific contrast agent (e.g. gadolinium ethoxybenzyl diethylenetriamine pentaacetic acid [Gd-EOB-DTPA]) has allowed the use of T1 mapping to accurately evaluate liver function [13–15], liver fibrosis [16], and FLLs [17]. However, these liver-specific contrast agents have low popularity and high economic cost in low- and middle-income countries, as they are not covered by health insurance in many countries. We aimed to compare the diagnostic value of DWI and B1-corrected T1 mapping with a non-specific gadolinium contrast agent (gadolinium diethylenetriamine pentaacetic acid) [Gd-DTPA]) for diagnosing FLLs.

## Methods

### Patients

This retrospective study was approved by the institutional review board of our hospital. The requirement for informed consent was waived due to the retrospective nature of the study.

From June 2018 to June 2020, a total of 215 patients with liver lesions who underwent abdominal MRI scans were recruited for the study. Patients with the following characteristics were excluded from the study: (1) received treatment before MRI examination (eight patients); (2) the diameter of FLLs was less than 10 mm (15 patients); (3) severe image artefacts that influence the measurements of ADC and T1 values; and (4) uncertainty of the lesion’s malignancy (14 patients). Consequently, 173 patients with 294 liver lesions were included in this study.

Surgery or biopsy was performed to confirm FNH, HCC, cholangiocarcinoma(CCA), HEM, and MET. In contrast, MRI was used to diagnose HEM, MET, and cyst. The diagnostic criteria for cysts are high SI on T2WI, low SI on DWI, and no enhancement after the injection of contrast agent with no change in cyst size for more than 6 months. For HEM, diagnostic criteria included high SI on T2WI, nodular enhancement on arterial phase, and delayed enhancement with no change in HEM size for more than 6 months. MET was identified according to the ring enhancement, perineural outflow effect in the delayed phase, and presence of a known primary tumour.

### MRI acquisition

All MR examinations were performed using a 3 T MRI system (Magnetom Skyra, Siemens Healthcare, Erlangen, Germany) with an 18-channel body coil combined with a 32-channel spine coil. All patients fasted 4–6 h before the MR scan and underwent breath-hold training. The patients were placed in a supine position for the MRI scan. The applied conventional sequences included coronal and axial fat-saturated T2W and axial T1W-volumetric interpolated breath-hold examination (VIBE)-Dixon. For contrast enhanced images, axial T1W VIBE-Dixon was performed 30, 65, and 100 s after the administration of Gd-DTPA (Gadopentetate dimeglumine, Consun, Guangzhou, China, 2.5 ml/s, 0.2 ml/kg) to acquire arterial, portal, and delayed phase images, respectively. Additionally, coronal T1W-VIBE-Dixon images were obtained for delayed phase images.

Before the injection of Gd-DTPA, single shot echo planar imaging DWI was performed under free breath condition with the following parameters: TR/TE = 5200/59 ms, FOV = 400 × 322 mm^2^, matrix = 134 × 134, slice thickness = 5 mm, slice distance = 1 mm, GRAPPA factor = 2, b value = 50 and 1200 s/mm^2^, bandwidth = 2332 Hz/Px, diffusion mode = 4 scan trace, and TA = 3 min and 10 s.

For T1 maps, the field map was first scanned for the B1 correction. Axial T1 maps of the liver were then acquired using the VFA method based on the VIBE sequence before and 120 s after the administration of contrast agent. The detailed parameters were: TR/TE = 5.05/1.83 ms, FOV = 380 × 306 mm^2^, matrix = 224 × 135, slice thickness = 4 mm, FA = 3° and 15°, CAIPIRINHA factor = 3, bandwidth = 300 Hz/Px, TA = 18 s.

### MR image analysis

Both ADC and T1 maps were inline calculated after the data acquisition. The ADC and T1 values of the lesions were measured on a workstation (Syngo.via, SIEMENS Healthcare, Erlangen, Germany) by depicting circular or elliptical regions of interest (ROIs); all ROIs were placed in the solid lesion. Additionally, necrosis, bleeding, fat, and visible vessels were avoided. Two radiologists (both with more than 8 years’ experience of liver MRI evaluation) performed the procedures and calculated ADC at three separate periods (before contrast enhancement), enhanced T1, and ΔT1% ([(Native T1 − Enhanced T1)/ Native T1] × 100%). Subsequently, the average values were considered the final results. The final diagnostic decisions of FLLs were made by the two radiologists.

### Statistical analysis

Statistical analyses were performed using SPSS (IBM Corp.,Armonk,NY,USA). The measured numerical data were presented as mean standard ± deviation. Additionally, inter-reader correlation was performed using the intraclass correlation coefficient (ICC). Chi-square test and one-way analysis of variance (ANOVA) were employed to evaluate the distribution of the participants’ sex, age, and lesion size. One-way ANOVA was used to compare the ADC, native T1, enhanced T1, and ΔT1% between benign and malignant FLLs. Statistical significance was set at *P* < 0.01. Further, the area under the curve (AUC) was determined, and the cut-off value was obtained to acquire the corresponding sensitivity and specificity. In addition, the differences in ADC and native T1 between cysts and haemangioma were compared. The correlation between ADC and native T1 and enhanced T1 and ΔT1% were analysed using the Pearson correlation coefficient.

## Results

Among the 173 patients included, 92 were male and 81 were female with a mean age of 52.1 ± 5.5 years (range: 28–75 years) (Table 1).

**Table 1:** Comparison of sex, age, and size distribution of different FLLs

	Cyst (n = 43)	HEM (n = 32)	FNH (n = 10)	HCC (n = 37)	CCA (n = 16)	MET (n = 35)	*P* value
Male/Female (n)	25/18	16/16	3/7	23/14	6/10	19/16	< 0.01
Age (years)	43.1 ± 3.5	46.3 ± 2.8	48.5 ± 1.3	49.7 ± 2.7	51.1 ± 3.0	61.1 ± 1.9	< 0.01
Size (mm)	25.3 ± 10.6	30.1 ± 20.1	28.5 ± 15.2	37.6 ± 18.5	22.3 ± 13.5	31.0 ± 14.3	< 0.01

*HEM* hemangioma; *FNH* focal nodular hyperplasia; *HCC* hepatocellular carcinoma; *CCA* cholangiocarcinoma; *MET* hepatic metastases; *FLL* focal liver lesion

The enrolled 294 FLLs (average size 29.5 ± 22.5 mm) included 150 benign lesions (male 44, female 41, average age 47.0 ± 2.1) with an average size of 27.3 ± 10.6 mm and 144 malignant tumours (male 48, female 40, average age 55.9 ± 2.5) with an average size of 30.8 ± 14.3 mm. Among the benign lesions, there were 43 patients with cysts, 32 with HEM, and 10 with FNH. Among the malignant lesions, there were 37 patients with HCC, 16 with CCA, and 35 with hepatic MET. (Table 1).

### Interobserver agreement

Satisfactory intrareader agreement was achieved. For all FLLs, the ICC of ADC, Native T1, and enhanced T1 were 0.92 (95% confidence interval [CI), 0.88 ~ 0.95), 0.98 (95% CI 0.95 ~ 0.99), and 0.99 (95% CI 0.97 ~ 0.99), respectively.

### DWI

**Fig. 1:**
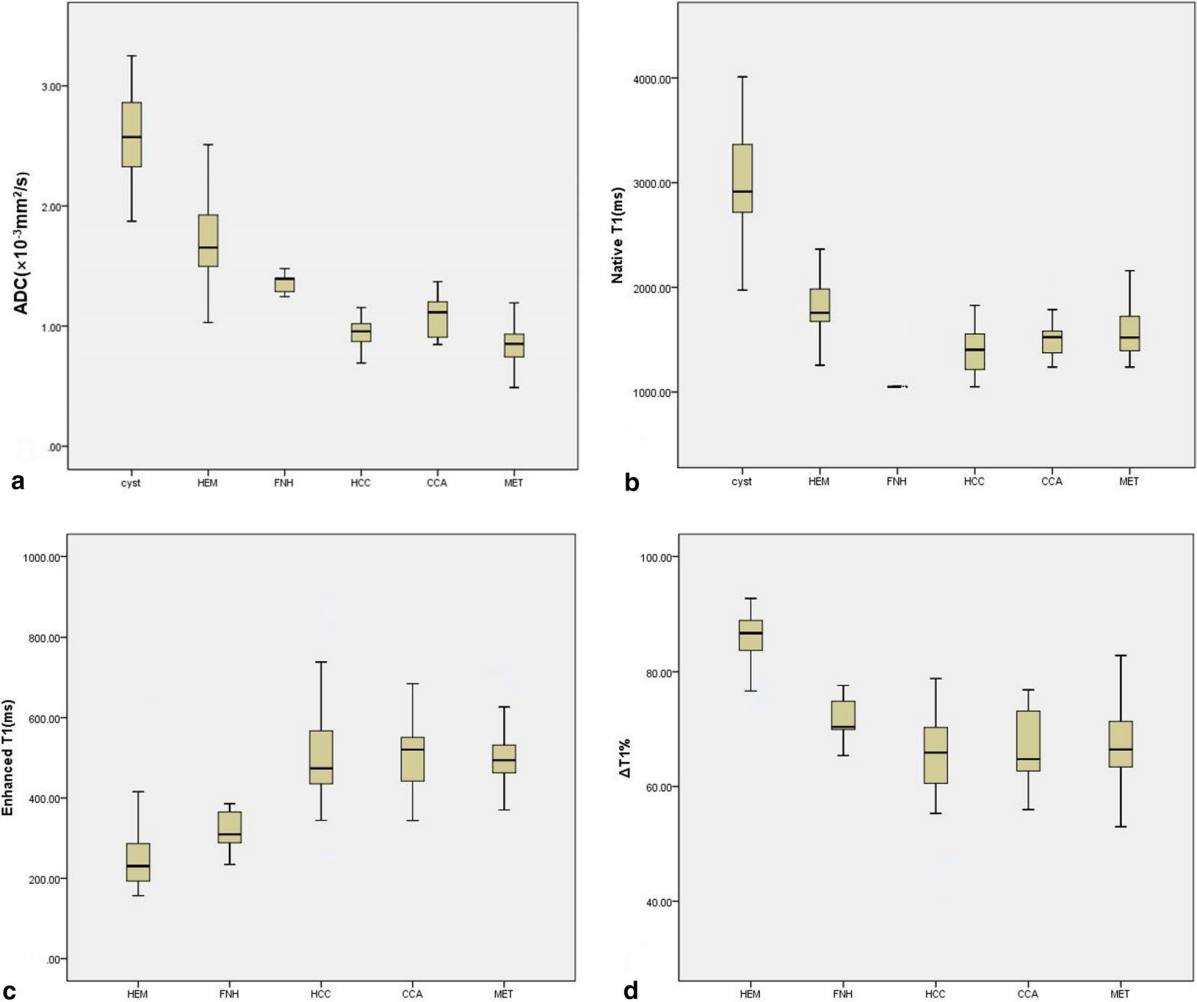
Box plot of ADC (**a**), native T1 (**b**), enhanced T1 (**c**), and ΔT1% **d** values of studied FLLs. HEM = haemangioma, FNH = focal nodular hyperplasia, HCC = hepatocellular carcinoma, CCA = cholangiocarcinoma, MET = hepatic metastases

The mean ADC value of malignant tumours (0.903 ± 0.21 × 10^–3^ mm^2^/s) was significantly lower compared to that of benign lesions (2.133 ± 0.36 × 10^–3^) (*P* < 0.01). Figure 1a indicates that the ADC of cysts was significantly compared to HEM, FNH, HCC, CCA, and MET (*P* < 0.01). The ADC of HEM was higher than that of FNH, HCC, CCA, and MET (*P* < 0.01). MET shows the lowest ADC value with no statistical difference from CCA and HCC (*P* = 0.84 and 0.89, respectively). Additionally, no significant difference was observed in the ADC value between HCC and CCA (*P* = 0.75).

Receiver operating characteristic (ROC) analysis showed that a cut-off value of 1.215 × 10^–3^ mm^2^/s could be used to identify benign and malignant lesions with a sensitivity and specificity of 0.975 and 0.952, respectively (Table 2). The ADC of HEM (1.647 × 10^–3^ mm^2^/s) was significantly lower compared to that of cysts (2.603 × 10^–3^ mm^2^/s) (*P* < 0.01); additionally, the ROC analysis showed that a cut-off value of 2.077 × 10^–3^ mm^2^/s can be used to differentiate between HEM and cysts with a sensitivity of 0.852 and a specificity of 0.812 (AUC 0.949, *P* < 0.01).

**Table 2:** Results of receiver operating characteristic (ROC) curve analysis for T1 mapping and DWI in differentiating benign and malignant FLLs

	AUC	95% CI	Cut-off value	Sensitivity	Specificity
Native T1(ms)	0.741	0.637–0.844	1653.525	0.797	0.702
Enhanced T1(ms)	0.979	0.950–1	339.2	0.911	0.976
ΔT1%	0.959	0.925–0.993	70.85	0.901	0.905
ADC (× 10^−3^mm^2^/s)	0.990	0.978–1	1.215	0.975	0.952

*AUC* area under the curve analysis; *CI* confidence interval; *ADC* apparent diffusion coefficient; *DWI* diffusion-weighted imaging; *FLL* focal liver lesion

### T1 mapping of FLLs

Malignant FLLs had significantly lower native T1 values (1487.3 ± 238 ms) compared to benign FLLs (2073.4 ± 345 ms) (*P* < 0.01).

For benign lesions, ANOVA indicates that the native T1 of cyst and HEM are significantly higher than those of HCC, CCA, and MET (*P* < 0.01); furthermore, the native T1 of cysts (3262.5 ms) is significantly higher than that of HEM (1824.0 ms) (*P* < 0.01). FNH had the lowest native T1, which was significantly lower compared to the other FLLs (*P* < 0.01).

For malignant lesions, HCC had the lowest native T1; however, no statistical significance was observed with CCA and MET (*P* = 0.76 and 0.82, respectively) (Fig. 1b).

ROC analysis demonstrated that the ideal cut-off value of native T1 was 1653.5 ms to distinguish between benign and malignant FLLs with a sensitivity and specificity of 0.797 and 0.702, respectively (Table 2). Additionally, the cut-off value of 2422.9 ms can be used to identify cysts and HEM with a sensitivity of 0.963 and a specificity of 0.972 (AUC 0.990, *P* < 0.01).

For the evaluation of enhanced T1 and ΔT1%, cysts were not included because they were non-enhanced lesions. The mean enhanced T1 value of benign FLLs was 268.7 ms, which was significantly lower compared to that of malignant tumours (mean value 486.0 ms, *P* < 0.01); in contrast, the ΔT1% of benign FLLs was significantly higher compared to that of malignant FLLs (*P* < 0.01). ANOVA indicated that HEM had the lowest enhanced T1 and the highest ΔT1%, which was significantly different from HCC, CCA, and MET (*P* < 0.01) (Fig. 1c and d). Furthermore, no statistical significance was observed regarding the enhance T1 of HEM and FNH (*P* < 0.88); however, the corresponding ΔT1% values were statistically different (*P* < 0.01). The enhanced T1 and ΔT1% of FNH were significantly different compared to those of HCC, CCA, and MET (*P* < 0.01). For malignant FLLs, HCC showed the lowest enhanced T1 value, with no statistical differences with CCA and MET (*P* = 0.87 and 0.95). Additionally, the ΔT1% of CCA has the lowest value but has no differences with HCC and MET (*P* = 0.92 and 0.94, respectively). The ROC analysis demonstrates that the ideal cut-off value of enhanced T1 was 339.2 ms to distinguish between benign and malignant FLLs with a sensitivity of 0.911 and a specificity of 0.976. In contrast, the ideal cut-off value of ΔT1% was 70.85% with a sensitivity of 0.901 and a specificity of 0.905 (shown in Table 2).

### Correlation between T1 Mapping and DWI

**Fig. 2:**
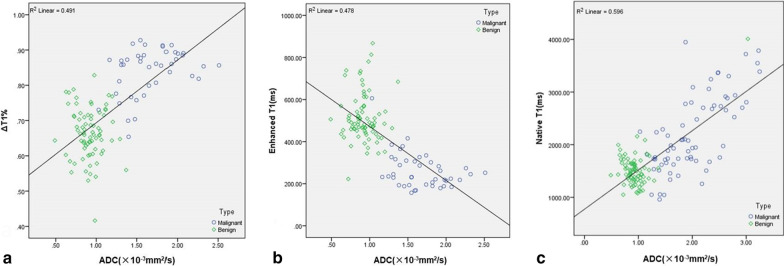
The correlation analysis between native T1 and ADC (**a**), enhanced T1 and ADC (**b**), and ΔT1% and ADC (**c**), indicating that native T1 and ΔT1% show significant positive correlation with ADC, and enhanced T1 shows significant negative correlation with ADC

**Fig. 3:**
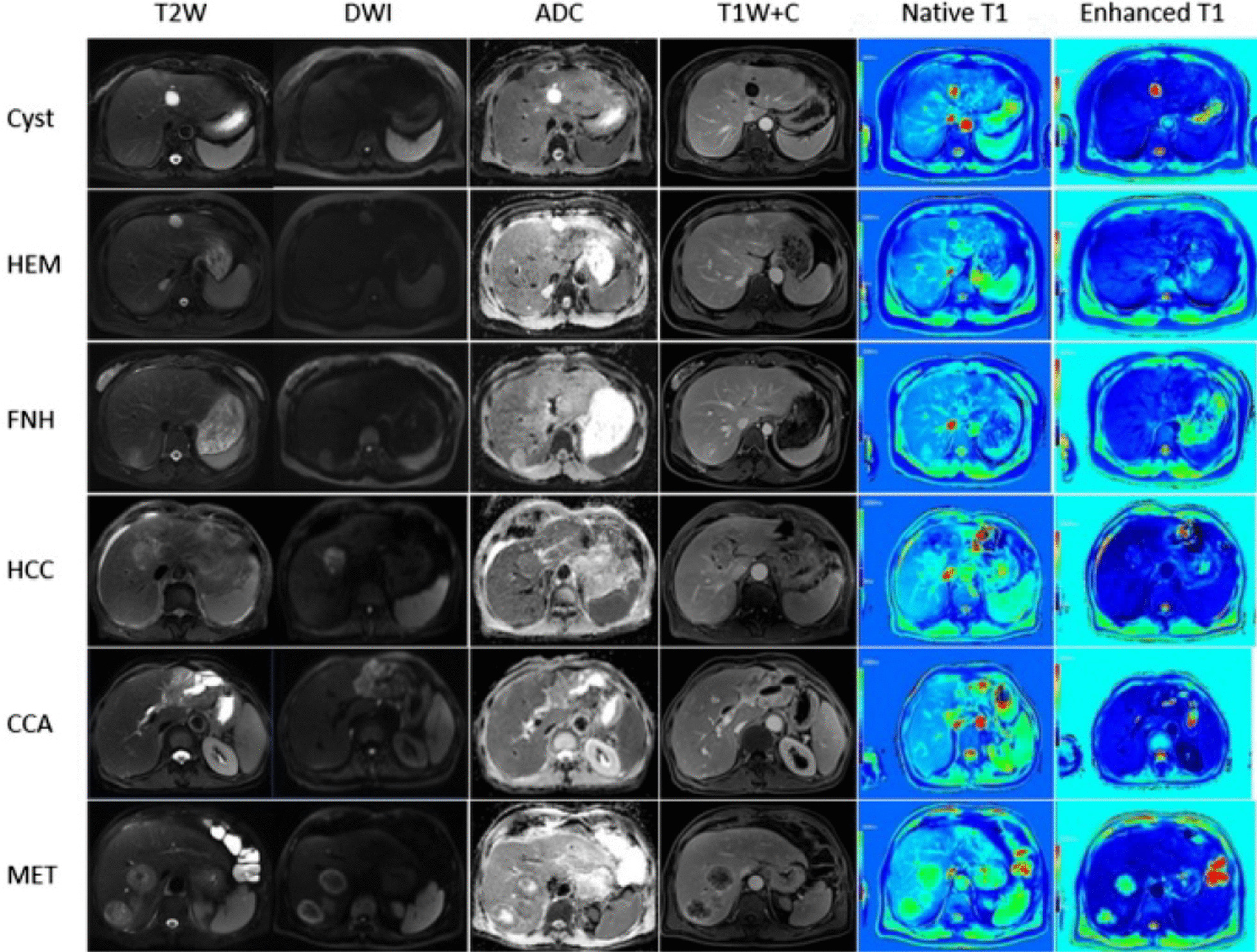
Representative images of patients with FLLs. Column 1: T2WI, Column 2: DWI (b = 1200 s/mm^2^), Column 3: ADC maps, Column 4: contrast enhanced T1W acquired 2 min after the injection of contrast agent, Column 5: native T1 mapping image, Column 6: enhanced T1 mapping images. Row 1: Cyst, Row 2: HEM, Row 3: FNH. Row 4: HCC. Row 5: CCA, Row 6: MET. HEM = haemangioma, FNH = focal nodular hyperplasia, HCC = hepatocellular carcinoma, CCA = cholangiocarcinoma, MET = hepatic metastases

The correlation analysis indicated a significant positive correlation between native T1 and ADC (r = 0.772, *P* < 0.01) (Fig. 2a), a significant negative correlation between enhanced T1 and ADC (r = -0.691, *P* < 0.01) (Fig. 2b), and a significant positive correlation between ΔT1% and ADC (r = 0.632, *P* < 0.01) (Fig. 2c). Representative images of all FLL-type lesions are shown in Fig. 3.

## Discussion

This study found that enhanced T1 acquired 2 min after the administration of Gd-DTPA showed the best performance for identifying benign and malignant FLLs; additionally, ΔT1% had a high potential for distinguishing between benign and malignant tumours. A previous study found that HCC had the lowest T1 decrease rate when enhanced T1 was obtained 20 min after administration of Gd-EOB-DTPA with different uptake rates in the delayed phase for different types of liver lesions [17]. Similarly, Yoshimura et al. showed that T1 mapping acquisition could be applied at the delayed phase to distinguish between MET and HEM [18]. The current study evaluated the T1 mapping obtained pre- and post- Gd-DTPA administration, a current widely used contrast agent for identifying different types of liver lesions. The enhanced T1 values were scanned 2 min after the administration of contrast agent, which is efficient for clinical practice, and the results were compared with the performance of DWI.

The current study employed the VFA technique for the acquisition of T1 mapping, which can similarly be achieved by other methods, such as Look-Locker or modified Look-Locker inversion recovery (MOLLI) [19, 20]. Compared with Look-Locker or MOLLI, VFA can measure the T1 value of the whole liver within a single breath hold, which allows maintenance of a high spatial resolution. However, VFA method is sensitive to B1 inhomogeneity, leading to an uneven distributed flip angle and T1 values, especially in high magnetic fields, such as 3 T. To overcome this problem, a previous study obtained a B1 field map prior to VFA sequencing to ensure the accuracy of T1 measurements [21]. Since not every equipment supplier can provide the B1 field-corrected sequence, and scanning B1 field calibration should be performed; however, it also needs a certain amount of acquisition time [22]. In the recent (21 December 2020) profile revision of the QIBA DCE-MRI Biomarker Committee call summary (https://qibawiki.rsna.org/images/0/0a/2020_12-07_QIBA_DCE-MRI_BC_Call_Summary-FINAL.pdf), because of the lack of literature and access to vendor-specific B1 mapping sequences, B1 correction was omitted as a DCE-MRI profile requirement. In the study of liver function and functional heterogeneity in patients with cirrhosis, T1 mapping imaging with corrected B1 inhomogeneity performed better than the uncorrected one [12]. Tadimalla et al. believe that B1 field correction may have a significant impact on the overall deviation and accuracy of individual subject-level measurement [23]. Therefore, the effect of B1 field correction on VFA T1 mapping imaging needs to be further confirmed. However, the equipment supplier used in this study provided the B1 field-corrected sequence, and B1 field correction was added in previous VFA T1 mapping imaging studies using equipment provided by the equipment supplier [20, 21, 24]. Therefore, In the present study, we also added B1 field mapping before T1 mapping to overcome the effect of B1 field inhomogeneity.

DWI has been used for the diagnosis of liver lesions [4–6] and grading of HCC [25, 26]. Moreover, advanced diffusion models, such as intravoxel incoherent motion [7], diffusion kurtosis imaging [8], and stretched exponential model [27], have been used to investigate liver diseases. Among them, ADC remains the most widely used and robust diffusion parameter in clinical practice. Namimoto et al. [28] reported the effectiveness of mean and minimum ADC in distinguishing between benign and malignant tumours, which was consistent with our findings. The ROC analysis indicated that an ADC cut-off value of 1.215 × 10^–3^ mm^2^/s can differentiate benign from malignant FLLs with an AUC, sensitivity, and specificity of 0.99, 0.975, and 0.952, respectively. A previous study [29] achieved excellent high sensitivity and specificity for ADC to evaluate FLLs; however, Mungai et al. [30] reported that approximately half of non-cystic lesions cannot be diagnosed with DWI. These contradictory findings may be due to different factors influencing DW image quality, such as respiratory movement, b value, spatial resolution, and field strength. In the present study, b values of 50 and 1200 s/mm^2^ were adopted to eliminate the T2 effect and improve the accuracy of the ADC calculation.

Both cysts and HEM showed high signal intensity in T2W images and can be identified using native T1 with a sensitivity and a specificity higher than 0.95; these values were better compared to those of DWI, enhanced T1, and ΔT1%. This study revealed that cysts have the highest native T1 and ADC values; these findings may be attributed to the high free water content of cysts and HEM [31]. However, HEM has lower native T1 and ADC values compared to cysts due to the presence of a fibrous septum, endothelium, and blood [32, 33]. The ROC analysis indicated that the AUC, sensitivity, and specificity of native T1 were better compared to ADC. Therefore, native T1 can be adopted as a non-enhancement method for the diagnosis of cysts and HEM; additionally, it has a shorter scan time compared to DWI, which benefits patients who are instructed to hold their breaths during MRI.

FNH showed the lowest native T1 value among all FLLs, which was consistent with a previous study [17]. The mean enhanced T1 of FNH was non-significantly higher compared to that of HEM. The ΔT1% of the FNH was significantly lower compared to that of HEM. FNH comprises multiple mononuclear cells with a diameter of 1 mm on a cell plate of normal liver cells and surrounds the enlarged artery [34]. Therefore, the SI of FNH on T1W is equal to or slightly lower compared to surrounding normal liver tissue. However, this study only included 12 FNH lesions; therefore, this finding needs to be validated by future studies employing a larger sample size. Additionally, we observed that the enhanced T1 of FNH was lower compared to that of HCC and was significantly different from CCA and MET. These findings were partially consistent with the results of Peng et al. [17] using Gd-EOB-DTPA who found that the enhanced T1 of FNH in the hepatobiliary phase was significantly lower compared to that of CCA and HCC.

Native T1 can be used to differentiate between HEM and MET, which is consistent with the findings of a previous study [18]. No significant differences were observed regarding the quantitative parameters between HCC, CCA, and MET. This may be due to the presence of complex components in malignant tumours, including haemorrhage and necrosis.

The ROC analysis revealed that ADC, enhanced T1, and ΔT1% had high sensitivities and specificities (all above 0.9); however, among them, ADC showed the highest values. Thus, T1 mapping remains an alternative despite the contamination of DWI image quality by unexpected factors, such as motion or imperfect fat saturation. Moreover, for benign tumours, native T1 showed better performance than ADC in distinguishing between cysts and HEMs. In this study, the diagnostic efficacy of enhanced T1 mapping was superior to that of ΔT1% and native T1, which is inconsistent with the results of Peng et al. [17], which indicated that ΔT1% acquired during hepatobiliary phase is the best parameter for evaluating FLLs. This disparity is mainly due to the differences in the type of contrast agent and operation method used in the two studies. The present study also reports that native T1 and ΔT1% have significant positive correlation with ADC, and enhanced T1 shows significant negative correlation with ADC.

This study has some limitations. First, pathology results were not obtained for all FLL patients; however, this limitation is inevitable because obtaining histopathological evidence is generally an uncommon practice when characteristic imaging features are observed in benign FLLs [17, 18]. Second, other liver lesions were not included in the current study, such as regenerative nodules, dysplastic nodules, and hepatic adenomas; future studies will be conducted to expand our database of FLLs. Third, the involved lesion size measured > 10 mm in diameter, mainly due to the limited spatial resolution of the DWI sequence. In future studies, small liver lesions will be focused on using T1 mapping technique.

## Conclusion

In summary, the diagnostic accuracy of the 3D T1 mapping acquired after the administration of Gd-DTPA was close to that of DWI, and native T1 showed a better performance than DWI in distinguishing between cyst and hemangioma. Therefore, T1 mapping is a potential noninvasive method for the detection of FLLs, and its diagnostic value for small liver lesions should be evaluated in future studies.

## Data Availability

The data that support the findings of this study are available from the corresponding author but restrictions apply to the availability of these data, which were used under license for the current study, and so are not publicly available. Data are however available from the authors upon reasonable request and with permission of the corresponding author.

## Abbreviations

ADC: Apparent diffusion coefficient
ANOVA: Analysis of variance
AUC: Area under the curve
CCA: Cholangiocarcinoma
CE-MRI: Contrast-enhanced magnetic resonance imaging
CI: Confidence interval
DWI: Diffusion-weighted imaging
FLLs: Focal liver lesions
FNH: Focal nodular hyperplasia
GD-DTPA: Gadolinium contrast agent
Gd-EOB-DTPA: Gadolinium ethoxybenzy diethylenetriamine pentaacetic acid
HCC: Hepatocellular carcinoma
HEM: Hemangiomas
ICC: Intraclass correlation coefficient
MET: Metastases
MOLLI: Modified Look-Locker inversion recovery
MRI: Magnetic resonance imaging
ROC: Receiver operating characteristic
ROIs: Regions of interest
SI: Signal intensity
VFA: Variable flip angle
VIBE: Volumetric interpolated breath-hold examination
